# Patient experience following iliac crest-derived alveolar bone grafting and implant placement

**DOI:** 10.1186/s40729-019-0200-8

**Published:** 2020-02-05

**Authors:** Cecilie G. Gjerde, Siddharth Shanbhag, Evelyn Neppelberg, Kamal Mustafa, Harald Gjengedal

**Affiliations:** 1 Department of Oral and Maxillofacial Surgery, Institute of Clinical Dentistry, University of Bergen, Årstadveien 19, 5009 Bergen, Norway; 2 Centre for Clinical Dental Research, Institute of Clinical Dentistry, University of Bergen, Bergen, Norway; 3 Department of Oral and Maxillofacial Surgery, Head and Neck Clinic, Haukeland University Hospital, Bergen, Norway; 4 Department of Prosthodontics, Institute of Clinical Dentistry, University of Bergen, Bergen, Norway

**Keywords:** Dental implants, Reconstruction, Quality of life, Bone graft, Iliac crest, Donor site morbidity, PROMs

## Abstract

**Background:**

The objective of this study was to assess patient-reported outcomes such as satisfaction and quality of life after advanced alveolar bone augmentation with anterior iliac crest grafting and implant treatment in orally compromised patients.

**Methods:**

This cross-sectional retrospective cohort study included 59 patients (29 women and 30 men) with major functional problems, who underwent advanced alveolar augmentation with autologous iliac bone grafts during a 10-year period (2002–2012).

The self-administered questionnaire included 36 validated questions related to (1) demographics, (2) perceived general and oral health, (3) donor site and hospitalization, (4) status of implants and/or prosthesis, and (5) oral health-related quality of life (OHRQoL).

**Results:**

Questionnaires were completed by 44 patients: 24 women and 20 men (response rate, 74.6%). Most patients reported good tolerance of the operative iliac bone harvesting (85%) and implant (90%) procedures. Post-operative pain at the donor site was reported by 38%, lasting 18.1 ± 16.1 days. An average of 4.3 ± 3.5 days of hospitalization and 20.2 ± 18.5 days of sick leave was reported. The overall satisfaction with prosthetic reconstruction was 90.5%. OHRQoL was reported with a mean Oral Health Impact Profile-14 (OHIP-14) score of 8.4.

**Conclusion:**

Favorable OHRQoL and satisfaction were reported after advanced reconstruction of alveolar ridges with iliac crest-derived grafting and implants in severely compromised patients. However, this treatment requires substantial resources including hospitalization and sick leave.

## Background

Insufficient alveolar bone volume, as a result of periodontal disease, trauma, congenital anomalies and/or resorption atrophy, often presents a clinical challenge for optimal placement of dental implants for prosthetic rehabilitation. In such cases, augmentation of alveolar bone, with either autologous bone, allogeneic, xenogeneic, or alloplastic biomaterials, is a prerequisite for placing implants in restoratively and esthetically acceptable positions.

Limited alveolar ridge defects are solved by local grafting. In cases of larger defects and extreme resorption, larger grafts are necessary. The most common donor site for large autologous bone grafts is the iliac crest, due to its accessibility, comparatively abundant bone volume, and high bone quality [1].

Autologous bone is still considered as a “gold standard” for alveolar reconstruction, according to systematic reviews [2–5]. Intra-oral donor sites, like mandibular ramus and symphysis, allow harvesting of limited volumes of autologous bone. The anterior iliac crest is the preferred extra-oral donor site for alveolar augmentation for larger bone volumes [1, 6, 7]. However, complications are reported, including pain, gait disturbance, hematomas, paranesthesia, and infections [8–15].

Traditionally, objective clinical variables, like the amount of bone gain (in millimeters) after augmentation, are reported as outcome measures after surgical procedures in clinical studies [16]. Patients’ experiences like patient-reported outcome measures (PROMs) have been increasingly used as a measure of treatment effect after medical and dental therapies [17, 18]. Importantly, these measures reflect the patients’ perceptions of the treatment outcome in addition to conventional clinical measures. Nowadays, Norwegian authorities address clinicians to include patients’ perspective in decisions regarding different treatment modalities [19]. It has been suggested that PROMs such as treatment satisfaction, perceived cost-effectiveness, and quality of life (QoL) may be more important and relevant to patients’ daily lives than objective clinical measures [16, 20]. Patient satisfaction is an important outcome measure, related to, although not synonymous with QoL, as satisfaction tends to reflect the process, rather than the outcome, of care [21]. Thus, an increase in the use of PROMs has been highlighted in dental implant research [22].

Health-related QoL (HRQoL) is a dynamic concept referring to an individual’s subjective assessment and perspective of current general health condition as well as functional, social, and emotional well-being [23, 24]. Most people regard oral health as important for QoL, and this is mediated through the concept of oral health-related QoL (OHRQoL) [25]. In this regard, OHRQoL is an important PROM in dental research, as oral health is an integral part of general health and well-being [26].

Different instruments to assess OHRQoL may be utilized to detect changes in physical, functional, and psychosocial impacts of oral disorders and have been validated for use in clinical studies [27–29]. The Oral Health Impact Profile-14 (OHIP-14) questionnaire is a widely used OHRQoL instrument [27]. It includes 14 questions covering seven domains of oral health and attempts to assess their impact on patients’ OHRQoL [30, 31]. OHIP-14 has previously been translated into Norwegian and used in a large study (*n* = 3538) with a calculated Norwegian national norm value [32]. Although previous studies have reported PROMs in relation to bone grafting [9, 33–42], to our knowledge, only one previous study has systematically assessed impact of donor site harvesting on OHRQoL, where (a) a post-operative lowering of OHRQoL was observed following bone grafting from both intra-oral and extra-oral sites and (b) iliac crest grafts compared to intraoral donor sites had a negative impact on postoperative QoL [37]. Moreover, to our knowledge, only one study has previously assessed the cost-effectiveness of autologous iliac crest grafting [43].

The aim of this study was to assess PROMs such as satisfaction and OHRQoL after advanced reconstruction of alveolar bone by anterior iliac crest-derived grafting and implant treatment.

## Methods

### Study population

This cross-sectional retrospective cohort study was based on records from all patients (*n* = 69) who underwent advanced alveolar augmentation with autologous iliac bone grafts at the Department of Oral and Maxillofacial Surgery, Haukeland University Hospital, Bergen, Norway, over 10 years (2002–2012). These patients were orally compromised with severe chewing problems as well as speech difficulties and had previously undergone several unsuccessful rehabilitation methods, prior to referral. At the time of this survey, seven patients had passed away, two had moved to unknown addresses, and one was hospitalized in a psychiatric institution. Thus, the study sample included 59 patients: 29 women and 30 men.

The Norwegian Committee for Medical Research Ethics (“REK,” Health Region West), acknowledged this study as a treatment quality control study.

### Treatment protocol—operative procedure

Bone graft surgeries were performed under general anesthesia and sterile conditions. Cortico-cancellous bone blocks were harvested from the anterior superior iliac crest. Reconstructions in the maxilla (*N* = 57) or mandible (*N* = 2) were performed in one operation by two teams using an onlay bone graft fixated with titanium micro-screws (1.5 mm Ø). The surgical procedure was performed according to the protocol commonly used at Haukeland University Hospital. In brief, the harvesting of autogenous bones from the anterior iliac crests started with a skin incision following the skin lines in a posterolateral direction starting from 3 to 4 cm medial to the iliac crests. The superior surfaces of the iliac crests are exposed after a sharp dissection through the periosteum following the crests. The dissections are performed with great attention to avoid laceration of the fascia lata. Both cortical and spongious bone are harvested. The donor sites are closed in layers with special attention to the first layer—the fascia lata. This layer is sutured close to avoid marrowbone bleeding. Activated vacuum drainages are positioned between the fascia lata and the muscles until the patients are mobilized. The skin incisions are closed with continuous intracutaneous resorbable sutures. All patients included in the study were hospitalized 2–3 days postoperatively. Patients received phenoxymethylpenicillin (1 g × 3) or clindamycin (300 mg × 3) for 5 days following the operation. Vacuum drainage at the donor site was used until the patient was mobilized the morning after surgery. Analgesics (paracetamol or non-steroid anti-inflammatory drugs) were prescribed 7–10 days postoperatively.

Implants were placed 4–6 months after the grafting procedure. The implant installations were performed by different oral surgeons (not in the hospital) and different implant systems were used. The implants installed into the augmented bone were allowed to heal for an additional 4–6 months before loading.

### Data collection

#### Medical records

The records of the original 69 patients were examined with regard to (1) grafting site (2), “graft-survival” determined by the ability to place implants in the grafted site(s) and (3) “implant survival” determined by the presence of functional implant-supported prostheses at the most recent follow-up. Reasons for implant failure were recorded when available.

#### Questionnaire

A self-administered questionnaire (Additional file 1) was sent by post to all 59 patients, together with an information leaflet about the survey, a return envelope with prepaid postage and an informed consent form. Reminder letters were sent after 2 and 4 weeks if no response was received.

The questionnaire contained 36 previously validated questions, which were categorized and related to (1) demographic and lifestyle, (2) perceived general and oral health, (3) donor site and hospitalization, (4) implant and prosthesis, and (5) OHRQoL (OHIP-14) (Table 1). Responses to questions in categories 1–2 were recorded as “yes/no” or graded on a 3- to 5-point Likert scale [44]. Category 3 included information on the duration of hospitalization and sick leave. Category 4 included information on “graft survival,” i.e., whether implants (and prostheses) were delivered in the augmented site(s), and “implant survival,” i.e., the presence or “loss/loosening” of any implants after surgery. OHRQoL was assessed using a Norwegian version of the OHIP-14 [32]. These 14 questions addressed seven domains of OHRQoL and their responses were graded on a 5-point Likert scale ranging from “at no time” (0) to “all of the time” (4) (Table 1).

**Table 1:** Summary of questions

Category	Response
Question	
(1) Perceived health-status	
General health	“Very good” to “bad”
Oral health	“Very good” to “bad”
Overall quality of life	“Excellent” to “bad”
(2) Lifestyle-related	
Smoking	“Yes,” “no,” or “sometimes”
Appetite	“Good” to “bad”
(3) Donor site-related	
Pain	“Yes” and “no”
Infection	“Yes” and “no”
Presence of a scar	“Yes” and “no”
Reduced sensitivity	“No” to “total loss of sensitivity”
Problems walking	“No” to “a lot”
Satisfaction	“Very satisfied” to “dissatisfied”
(4) Implant-related	
Intraoral pain	“No” to “strong pain”
Installation of implants and prosthetic	“Yes”, “no” or “just implants”
Loss of implants	“Yes” and “no”
Satisfaction with prosthesis	“Very satisfied” to “dissatisfied”
(5) OHIP-14	“At no time” to “all of the time”

### Statistical analysis

Data were anonymized and analyzed using SPSS v 24 (SPSS Inc., Chicago, IL, USA). Descriptive analyses were applied. Statistical significance was set at 5% level.

## Results

The final sample consisted of 44 patients that responded and completed the questionnaire, giving a response rate of 74.6%: 24 women and 20 men, mean age of 61.2 years ± 13.1 (range 27–82 years). The mean time from augmentation surgery until completing the questionnaire was 7.8 years ± 2.65 (range 1.9–12 years).

Summary of demographic and lifestyle-related data is presented (Table 2).

**Table 2:** Patients’ demographic and lifestyle-related data

Variable	Frequency	
	*N* or Mean ± SD	%
Patients		
Female	24	54.5
Male	20	45.5
Age (years)	61.16 ± 13.10	
Age at operation	53.73 ± 13.07	
Time from augmentation to completing questionnaire (months)	93.55 ± 31.75	
Civil status		
Married	30	68.2
Single	11	25.0
Widow(er)	3	6.8
Housing		
Alone	12	27.3
With another person	23	52.3
> two persons	9	20.5
Education		
Up to primary	7	11.3
Up to secondary	23	53.5
“Artium”	1	2.3
High school	9	20.9
University	3	7.0
Smoking		
Yes	8	19.0
No	33	78.6
Sometimes	1	2.4
Cigarettes/day	13.65 ± 7.22	
Years of smoking	26.52 ± 11.63	

### Descriptive findings

#### Health-related PROMs

Most patients reported “good” or “very good” levels of general health (81.4%), oral health (83.7%), and overall quality of life (90.7%). Less than 5% reported “bad” levels for either of these variables. Most patients reported better general (86%) and oral health (78%) after treatment. Only two patients (4.7%) reported their oral health to be worse after treatment.

#### Donor site- and hospitalization-related PROMs

Most patients (85.4%) were satisfied with the hip surgery procedure. Pain at the donor site was reported by 38% of patients, lasting for an average of 18.1 ± 16.1 days and measuring 43.6 ± 27 on the VAS (0–100) scale. Only two patients (4.7%) reported post-operative infection at the donor site. Scar formation on skin (hip) was reported in 49% of patients, by majority esthetically acceptable (90.4%). Four (9.5%) and two (4.7%) patients reported “a little” or “a lot” of reduced sensitivity at the donor site, respectively. Three patients (7.3%) reported problems in walking (Table 3). The average time of hospitalization was 4.3 ± 3.5 days and sick leave 20.2 ± 18.5 days.

**Table 3:** Patient-reported outcomes

Question	Response	Frequency
Oral health	Very good/good	81.8%
Quality of Life	Very good/good	90.9%
General health	Very good/good	81.8%
Pain after hip operation	Excessive	35.0%
Satisfaction hip operation	Very	85.7%
Post op infection in hip site	No	95.3%
Visible scar on hip	Yes	48.8%
Acceptable scar	Yes	20 of 21^a^
Reduced sensibility on hip site	No	86.0%
Problem walking	No	92.9%
Augmented bone block still present	No	6.8%
New augmentation	Yes	1 of 4^a^
Oral pain after augmentation	No/some	83.3%
Implant/teeth in augmented bone	Yes	90.9%
Lost implants	Yes	28.6%
Time lost after installation	0–3 months	42.9%
	7–12 months	28.6%
New implants installed	Yes	8 of 11^a^
Satisfaction with implant-retained teeth	Very satisfied/satisfied	90.5%

^a^Incomplete or missing data

#### Implant-/prostheses-related PROMs

Most patients (*n* = 40, 90.9%) reported to have implants placed and received prostheses in the augmentation site(s). This was interpreted as graft survival, indicating a graft survival rate of 90.9% on the patient level. Two patients received implants, although without further prosthetic rehabilitation. Implants could not be installed in two patients. However, 29.3% of patients reported “loosening or loss” of implants in the post-operative period (1 year), indicating an implant survival rate on the patient level of 70.7%, and most patients (8 out of 11) received new implants.

No pain was reported in 39 patients (82.9%) following implant surgery and a majority of patients (90.2%) were satisfied/very satisfied with the implant therapy overall and in terms of overall satisfaction with teeth (90.5%).

The correlation analyses performed did not show a significant correlation between the complications at the donor site and implant loss (Table 4).

#### OHRQoL

The mean OHIP-14 score (Table 5) was 8.4 ± 9.7 (range 0–56) in 44 patients of whom 35 patients scored 14 or less. Nine patients scored a total sum of 1 [1], i.e. “hardly ever” impact on any single item and “at no time” on the remaining 13 items. The functional limitation domain had the highest score (2.34) and the social disability domain the lowest score (0.61).

**Table 4:** Correlation analyses

Outcome variables	Correlations	Spearman’s rho	*P* value
OHRQoL	Oral health compared	0.596	< 0.0001
	General health now	0.369	0.014
	General health compared	0.412	0.005
	Implants placed/teeth installed	0.317	0.036
	Lost implants	− 0.372	0.015
	Smoking	− 0.334	0.005
	Speaking	0.572	< 0.0001
	Chewing	0.375	0.014
Implants placed	General health	− 0.314	0.038
	Oral pain post op	0.334	0.031
	Oral health	0.305	0.044
	General health compared	0.314	0.038
	Satisfaction hip operation	− 0.439	0.004
	OHRQoL	0.317	0.036
Lost implants	General health	− 0.328	0.034
	QoL	− 0.342	0.027
	OHRQoL	− 0.372	0.015
	Satisfied teeth	− 0.328	0.034

**Table 5:** Summary of OHIP-14 (*N* = 44 and response range 0–8)

OHIP domain	Minimum	Maximum	Mean	SD
Functional limitation	0	7	2.34	1.70
Physical pain	0	7	1.16	1.51
Psychological discomfort	0	8	1.64	2.27
Physical disability	0	8	0.75	1.77
Psychological disability	0	8	1.18	2.11
Social disability	0	8	0.61	1.40
Handicap	0	8	0.70	1.71
Total	0	64	8.4	9.7

## Discussion

An important finding in this study is that a majority of patients were very satisfied after iliac crest-derived alveolar bone grafting and implant therapy. Although 90% of the patients in our study had successful bone grafting, only 70.1% reported implant survival together with prosthetic rehabilitation after 1 year. These figures are lower than those reported in previous studies [2, 3, 9]. A review by Chiapasco et al. showed that the mean graft failure in 16 studies was 1.6% and partial loss of graft of 3.3% [45]. The same review showed that the overall survival rate of dental implants in transplanted bone was 87%. However, it must be kept in mind that the patients in our study were orally compromised and very challenging to reconstruct. Moreover, the patients in our study did not report on the number of implants lost, and we do not have reliable records of the exact number of implants each patient had got installed. This could indicate differences in survival on implant or patient levels—a variable of clinical importance as the number of lost implants may be higher.

Another important finding is that patients reported to tolerate the augmentation procedure well; 85% of patients were satisfied with the hip operation (performed under general anesthesia), comparable to a previous report [46]. However, 40% of the patients reported pain for 18 ± 16 days after augmentation, which is in accordance with other studies [37, 46] and which should be considered during the treatment planning of patients scheduled to received iliac crest-derived bone grafts [33]. Two patients reported infection at the donor site. All operations were performed by a strict sterile regime and protocol at the university hospital.

The level of OHRQoL reported by the patients was favorable with an OHIP-14 value of 8.4. In a previous study, Dahl et al. reported an OHIP-14 score of 4.1 in the Norwegian adult population (2441 patients), with 35% of the sample reporting “no oral health problems” [32]. If the study sample in the study of Dahl et al. is considered to be representative of the general population, patients in our study reported poorer OHRQoL than the general population. Thus, even though the participants in this study report good oral health and better than before operation on the single questions, they still report having problems related to their oral condition. This is to be expected as the patients in our study were orally compromised before augmentation with almost no alveolar ridge to retain or support a prosthetic construction. Since the patients had extensive alveolar bone loss rendering them orally handicapped, any improvement in function would be likely to have a positive impact on satisfacation and OHRQoL. However, it is difficult to relate their reported level of OHRQoL to the augmentation and implant installation per se, as this was performed up to 12 years prior to completing the questionnaire (mean 7 years and 10 months). So, patients’ present oral situation with fixed teeth could/may alter the “reference” for the patients regarding OHRQoL. However, we cannot reliably ascribe the level of OHRQoL to the treatment performed years ago, since we have no such data either before or soon after the prosthetic rehabilitation, and therefore, cannot estimate the influence the effect of response shift on the study outcomes. Previous reports show a significant influence of implant-retained prosthetic treatment on OHRQoL, but these reports are based on before-and-after registrations [47].

Patients in our study reported satisfaction with the augmentation and implant installation, and as these patients were orally compromised before the operation, their satisfaction with getting fixed teeth most likely improved their perceived oral health condition. This might also, in part, explain why they reported good OHRQoL. Thus, our findings indicate that a majority of patients tolerate the augmentation and implantation procedures very well and with minor long-term sequelae.

The treatment protocol described in this study, i.e., advanced bone reconstructions under general anesthetics, hospitalization, and sick leave, is considered expensive in a public health services. In the present study, an average of 4.3 days of hospitalization and 20.2 days of sick leave was reported, which is costly for the health service and inconvenient for the patient [33, 43]. When comparing iliac bone graft as a treatment to bone substitutes, a previous study clearly demonstrated that iliac bone graft procedure demands more resources and more than three times the costs of bone substitutes [43]. Although the patients reported good satisfaction and OHRQoL after iliac bone grafting, this treatment is demanding for patients as well as health services, indicating the need for alternative treatment modalities [37, 43, 46].

## Conclusions

Favorable OHRQoL and satisfaction were reported after advanced reconstruction with iliac crest-derived grafts and implant treatment in orally compromised patients. However, this treatment requires substantial resources including hospitalization and sick leave.

## Supplementary information

**Additional file 1.** A self-administered questionnaire.

## Data Availability

The datasets used and/or analyzed during the current study are available from the corresponding author on reasonable request.

## Abbreviations

HRQoL: Health-related quality of life
OHIP-14: Oral Health Impact Profile-14
OHRQoL: Oral health-related quality of life
PROMs: Patient-reported outcome measures
QoL: Quality of life
