# Supplementary material for: Patient experience following iliac crest-derived alveolar bone grafting and implant placement
Source: Int J Implant Dent. 2020 Feb 5;6:4. doi: 10.1186/s40729-019-0200-8 (PMC7000591; doi:10.1186/s40729-019-0200-8)
Supplement: Supplementary file 1 — Additional file 1. A self-administered questionnaire. [file 40729_2019_200_MOESM1_ESM.docx]

Dato:

ID nr.

SPØRSMÅL ETTER BENOPPBYGGING MED BEN FRA HOFTEN

DIN TANNHELSE NÅ (HELE MUNNHULEN)

*1. Hvordan synes du (helse) forholdene i munnen din er?*

1  Meget god

2  God

3  Ikke helt god

4  Dårlig

*2. Hva var hovedårsaken til ditt siste tannlege besøk?*

1  Et akutt tannproblem

2  Rutinekontroll, ble innkalt av tannlegen

3  Rutinekontroll, bestilte selv time

*3. Hvor mange ganger har du vært til tannlegen i løpet av de siste 5 årene?*

1  5 ganger eller mer

2  3-4 ganger

3  1-2 ganger

4  Har ikke vært til tannlegen (de siste 5 årene)

*4. Hvor ofte rengjør / pusser du tennene*

1  Aldri

2  En gang i blant

3  En gang for dagen

4  Flere ganger for dagen

GENERELL INFORMASJON

*Livskvalitet er avhengig av så mange forhold, slik som familierelasjoner, økonomi, levekår, vennskap, framtidsutsikter, helse, politiske forhold, osv.*

*5. Stort sett, vil du si at din livskvalitet er:*

1  Utmerket

2  Meget god

3  God

4  Nokså god

5  Dårlig

*6. Alder (i fylte år) ________________*

*7. Kjønn*

1  Kvinne

2  Mann

*8. Hvordan er din generelle helsetilstand nå?*

1  Svært god

2  God

3  Verken god eller dårlig

4  Ikke helt god

5  Dårlig

*9. Hvordan er appetitten din?*

1  God

2  Verken god eller dårlig

3  Dårlig

*10. Sivilstand*

1  Gift / samboer

2  Ugift

3  Enke/ enkemann

*11. Har du, eller har du hatt:*

*(svar på hvert enkelt spørsmål)*

alder

1. gang

(1) ja (2) nei

1. Hjerteinfarkt   _____

2. Angina pectoris

(hjertekrampe)   _____

3. Hjerneslag/hjerne-

blødning (”drypp”)   _____

4. Astma   _____

5. Diabetes (sukkersyke)   _____

6. Høyt blodtrykk   _____

7. Beinskjørhet   _____

8. Giktfeber   _____

9. Smittsomme sykdommer,

f.eks. Hepatitt   _____

Annet:

*12. Sammenlignet med før operasjonen, hvordan vil du si at din helse stort sett er nå?*

1  *Svært god*

*2  God*

*3  Verken god eller dårlig*

*4  Ikke helt god*

*5  Dårlig*

(1)ja (2)nei (3)av og til

*13. Røyker du selv:*

Sigaretter daglig?

Sigarer/sigarillos daglig?

Pipe daglig?

*14. Hvis du røker daglig (eller nesten daglig) nå, eller har røkt tidligere:*

Hvor mange sigaretter røyker/røykte

du vanligvis daglig? _______

Hvor mange år til sammen

har du røykt daglig? _______

*15. I hvilken grad er du plaget med*

en god

del

svært

mye

aldri litt

Tørr i munnen?

Tørr i munnen

når du spiser?

Kjeveleddsproblemer?

At maten generelt

smaker lite?

*16. Hvor mange personer bor det i husstanden?*

1  Bor alene

2  To

3  Flere enn to

*17. Yrke / tidligere yrke*

……………………………………………………...

*18. Hva slags utdannelse har du (din høyeste full-førte utdannelse)?*

Kryss bare en gang

1  Ingen utdannelse etter *grunnskolen* (folkeskole / framhaldsskole / ungdomsskole)

2  Realskole / fagskole / yrkesskole / handels-skole

3  Artium

4  Høyskole / lavere grad universitet utdannelse (grunn - / mellomfag)

5  Universitetsutdannelse (hovedfag, embetseksamen, etc.)

6  Annen (spesifiser)

………………………………………………...

ETTER OPERASJONEN

*De neste spørsmålene handler om hvordan du følte deg etter operasjonen med beintransplantasjon*

*19. Hadde du smerter i hoften*

Nei

Ja

Antall dager:

Antall timer:

På denne linjen kan du krysse av hvor intens du følte smerten var

Ingen Intens

Smerte smerte

*20. Infeksjon i såret etter operasjonen på hoften*

Nei

Ja

Varighet i dager:_______

Behandlet med antibiotika

(sett ring rundt svaret): Ja Nei

*21. Synlig arr på hoften nå*

Nei

Ja

Akseptabelt

Skjemmende

*22. Nedsatt følsomhet nå i sårområdet på hoften*

Nei

Litt

Mye

Ingen følelse i området

*23. Problemer med å gå nå pga operasjonen i hoften*

Nei

Litt

Mye

*24. Er du fornøyd med operasjonen på hoften*

Nei

Litt fornøyd

Passelig fornøyd

Helt fornøyd

*25. Hvor mange dager var du innlagt på sykehuset i forbindelse med denne operasjonen:*

________dager

*26. Hvor mange dager var du sykemeldt etter operasjonen:*

________dager

*27. Hadde du smerter i munnen etter operasjonen med benoppbygging*

Nei

Litt

Mye

Sterke smerter

*28. Har du fått implantat og tenner der det ble gjort benoppbygging*

Ja

Nei

Bare implantat

*29. Har noen av implantatene løsnet og falt ut*

Nei

Ja

Dersom Ja:

Har fått nye implantat senere

Har ikke fått nye implantat senere

TILFREDSHET MED TANNERSTATNINGENE

*23. Hvordan har du stort sett vært fornøyd med tennene som er festet til implantatene?*

1  Meget godt fornøyd

2  Godt fornøyd

3  Ikke helt fornøyd

4  Misfornøyd

*31. Hvordan er du fornøyd med tennenes utseende?*

1  Meget godt fornøyd

2  Godt fornøyd

3  Ikke helt fornøyd

4  Misfornøyd

*32. Hvordan er du fornøyd med tyggingen?*

1  Meget godt fornøyd

2  Godt fornøyd

3  Ikke helt fornøyd

4  Misfornøyd

*33. Hvordan er det vært å snakke med de nye tennene?*

1  Meget godt fornøyd

2  Godt fornøyd

3  Ikke helt fornøyd

4  Misfornøyd

*34. Hvordan er det å pusse de nye tennene*

1  Går veldig fint

2  Går greit

3  Litt vanskelig

4  Veldig vanskelig

*35. Sammenlignet med før operasjonen, hvordan anser du stort sett tannhelsen/forholdene i munnen din for å være*

1  Svært god

2  God

3  Verken god eller dårlig

4  Ikke helt god

5  Dårlig

*36. Har du i det siste året hatt følgende problemer eller ubehag på grunn av dine tenner eller proteser eller på grunn av andre forhold i munnen?*

|  | |  | | Ikke opplevd | | Sjelden | | | | Av og til | Ganske ofte | Ofte |
| --- | --- | --- | --- | --- | --- | --- | --- | --- | --- | --- | --- | --- |
| 1 | | Vanskeligheter med å uttale ord eller lage spesielle lyder | |  | |  | | | |  |  |  |
| 2 | | Endret/dårligere smakssans | |  | |  | | | |  |  |  |
| 3 | | Smerter eller vondt i tennene, munnen | |  | |  | | | |  |  |  |
| 4 | | Opplevd at mat har gitt ubehag | |  | |  | | | |  |  |  |
| 5 | | Følt deg usikker pga tenner, forhold i munnen, proteser | |  | |  | | | |  |  |  |
| 6 | | Følt deg spent eller stresset pga tenner, forhold i munnen, proteser | |  | |  | | | |  |  |  |
| 7 | | Hatt en dårlig kost/kostsammensetning | |  | |  | | | |  |  |  |
| 8 | | Måttet avbryte måltider | |  | |  | | | |  |  |  |
| 9 | | Hatt problemer med å slappe av | |  | |  | | | |  |  |  |
| 10 | | Følt deg sjenert | |  | |  | | | |  |  |  |
| 11 | | Vært irritabel overfor andre mennesker | |  | |  | | | |  |  |  |
| 12 | | Hatt vanskeligheter med dine vanlige gjøremål | |  | |  | | | |  |  |  |
| 13 | | Følt at livet i sin alminnelighet var mindre tilfredsstillende | |  | |  | | | |  |  |  |
| 14 | | Ikke kunnet fungere i hverdagen | |  | |  | | | |  |  |  |
|  |  | |  |  |  | |  |  |  |  |  |  |
